# The Folkestone Health Congress

**Published:** 1921-07-02

**Authors:** 


### THE FOLKESTONE HEALTH CONGRESS.

The thirty-second Congress of the Royal Sanitary Institute, which was held at Folkestone from June 20 to 25, provided opportunity for the discussion of many of the problems which are urgently presenting themselves in connection with the organisation of the health services of the country. The Earl of Radnor in his presidential address struck the chord which appealed to all present when he urged the full development of those services free from the shackles of State control. Particularly in regard to hospitals and the nursing service did he press for the maintenance of the voluntary system. If that is destroyed the value of all the health work will to a great extent be destroyed also. He hopes the Ministry of Health and local health authorities will make the finest possible use of the voluntary agencies. Voluntary and State authorities must come closer together in the work which all desire to see carried out, and petty jealousies must disappear.

Out of the large number of papers which were read on the subjects covered by the various sections, viz.: (*a*) Sanitary Science and Preventive Medicine; (*b*) Engineering and Architecture; (*c*) The Hygiene of Maternity and Child Welfare, including School Hygiene; (*d*) Personal and Domestic Hygiene; (*e*) Industrial Hygiene, it is only possible to refer to a few. In (*a*) a very interesting group of papers was provided by Dr. W. J. Tyson, M.D., F.R.C.P., F.R.C.S., on "The Relationship of Hospitals to Preventive Medicine," by Mr. E. J. Martin Lobb, M.B., and Dr. C. Ponder on "The Hospital as the Primary Health Centre in Relation to a Health Service," from the standpoint of the general practitioner and the county health officer respectively, and by Dr. J. Middleton Martin on "The Gloucestershire Scheme for the Extension of Medical Services," which has already been described in these columns. Dr. Tyson discussed the means for the provision for everyone of the best modern treatment—in other words, hospital treatment—and indicated the desirability of insurance; he also pressed for payments by Approved Societies. A "Domesday Book" of England's convalescent accommodation was urged by Mr. Martin Lobb, with the assurance to every hospital, according to its size, of a fraction of the available convalescent bed accommodation, and the utilisation for the same purpose of vacant beds in Poor-law infirmaries. Mr. E. T. Hall's paper on "The Design of Hospitals" indicated the desiderata in building a general hospital, one for infectious disease, and sanatoria and hospitals for consumption. He made a point of the provision in every hospital of a chapel for divine service, and stated that the standard of the Ministry of Health of bed space in Poor-law infirmaries has been shown to be insufficient as a minimum for curative purposes; the space he favours is 8 feet wall space and about 100 superficial feet, except where there is a medical school, in which case 9 to 10 feet wall space and 117 to 135 superficial feet is allowed.

Cost of Public Health Work.

Dr. W. Allen Daley, M.O.H., Blackburn, gave some interesting information derived from the figures of expenditure of the departments of medical officers of health of county boroughs during 1919-20. These figures show variations in the cost of health work of from 2s. 3d. to 9s. 4d. per head of the population, and from 3⅝d. to 2s. 6d. per £ of rateable value, though the actual payments by the ratepayers are somewhat less owing to the Government paying 50 per cent. of the cost of anti-tuberculosis, maternity, and child-welfare and school medical service work, and 75 per cent. of the cost of measures taken against venereal diseases. He found that of every £100 expended on health work £26 is spent on infectious-disease work, mostly hospital maintenance, £30 on anti-tuberculosis work, £13 on maternity and child welfare, £5 on venereal diseases, £9 on school medical services, £2 on food control, and £15 on administration and general sanitation. Is the present allocation the best possible? And in this connection Dr. Daley remarked on the influence on public health expenditure of popular opinion. In his view a small expenditure on health visitors and schools for mothers undoubtedly produces splendid results, but when a little more is desired and hospitals are built the expenditure rises by leaps and bounds.

Need for Combination and Co-operation.

In the conference on Health Visitors Miss Gertrude Tuckwell urged the value of combination for the sake of the work and the worker. Miss Elizabeth Wyatt on the same lines appealed for closer co-operation between health visitors, district nurses, school nurses, and midwives; such co-operation should minimise expenditure and overlapping, give greater efficiency in the work, and conduce to better feeling between the workers and the mothers in the homes. Miss Wyatt outlined a constructive scheme to prevent overlapping and undue expenditure which would divide regions into workable areas, with a fully trained nurse in each to act as nurse, midwife, health visitor, and school nurse. These areas should be grouped into districts, each with its district superintendent, who would act as inspector of midwives and district nurses and supervise the health visiting and school nursing. Miss Weir (St. Helens), in her paper on "The Trained Nurse in Public Health," brought forward cogent reasons for the employment of the trained nurse as health visitor in preference to the health visitor who is the product of a special course of study made up of a few months here and a few months there. Every day, she urged, we hear that it is wrong to separate treatment and prevention in the education of medical men. Do not the same reasons hold good in the training of a health visitor? In ward management, too, the trained nurse learns to consider the fact that she is one of a community and must exert herself for the common good. Training in hospital should continue for four years, in the last of which the trained nurse should attend lectures outside the hospital, work in the public health department of the town, and act as staff nurse in her ward; she should also obtain the health visitor's certificate, afterwards entering a maternity hospital to take the C.M.B. certificate if not obtained at her training-school. She should then be attached to a public health department for six months as a probationer health visitor for a living wage, under the supervision of a trained health visitor.

Mention must not be omitted of the papers in the Industrial Hygiene Section by Mr. D. R. Wilson on "Some Effects of Environment on Efficiency and Safety" (based on the work of the Industrial Fatigue Research Board); by Mr. Robert Hyde, Director of the Industrial Welfare Society, on "The Organisation of Welfare Schemes"; and by Mr. E. Holden on "Welfare—a Path to Industrial Peace." The latter rightly said that the panacea for the removal of discontent is the full life. This can only be attained by an educated experience. And he reminded his hearers that "the true greatness of States lies not in territory, revenue, population, commerce, crops, or manufactures, but in immaterial or spiritual things. . . . With nations as with individuals none but moral supremacy is immutable and for ever beneficent."

